# Might salicylate exert benefits against childhood cancer?

**DOI:** 10.3332/ecancer.2010.156

**Published:** 2010-01-19

**Authors:** G Morgan, JI Johnsen

**Affiliations:** 1 Fellow of the Royal Institute for Public Health, National Health Service for Wales, Wales, UK; 2 Childhood Cancer Research Unit, Department of Woman and Child Health, Karolinska Institutet, Stockholm, Sweden

## Abstract

Childhood cancers are a broad range of diseases. Research on the chemopreventive potential of non-steroidal anti-inflammatory drugs, such as aspirin (acetylsalicylate) has yet to be fully directed towards childhood cancers. A ***prima facie*** hypothesis on salicylate and childhood cancer would therefore be based on several factors. Firstly, salicylate inhibits the production of inflammatory prostaglandins, which have been shown to stimulate the growth of cancer cells. Secondly, salicylate inhibits the growth of cancer cells in pre-clinical models. Thirdly, salicylate is a natural component of fruits and vegetables so it is consumed within the diet. Further research, of which some possibilities are identified, is recommended.

## Introduction

Aspirin, acetylsalicylate, belongs to a class of medicines known as non-steroidal anti-inflammatory drugs (NSAIDs). These medicines are widely used for the treatment and alleviation of pain, inflammation and fever. However, undesirable effects, such as gastrointestinal irritation and an increased risk of cardiovascular events, limit the use of these medicines [1].

In low doses of 75–150 mg/d, aspirin is used to reduce the risk of cardiovascular disease [2]. There is also a growing evidence-base that aspirin and other NSAIDs might reduce the risk of developing a wide range of cancers [3]. Hypotheses underpinning this possible benefit are that NSAIDs might exert chemopreventive effects during the process of cancer development, so-called multistage carcinogenesis [4]. One contributory effect is believed to be due, at least part, to the inhibition of cyclo-oxygenase (COX) enzymes, leading to the reduced production of pro-inflammatory eicosanoids, like prostaglandins [4]. COX-1 is expressed constitutively whilst COX-2 is induced during carcinogenesis, leading to high levels of prostaglandin E_2_ production that supports cancer growth. In addition, aspirin and other NSAID have also been shown to directly inhibit the growth of cancer cells through COX-2-independent mechanisms and by inhibition of angiogenesis [4]. Furthermore, salicylate, which is the principle active metabolite of aspirin in humans, has been shown to induce programmed cell death, apoptosis, in several cancer cell lines [4].

Childhood cancers are a broad range of diseases that are rare everywhere in the world [5]. In so-called developed countries, however, childhood cancers are the commonest cause of disease-related deaths in childhood, carrying with them great emotional and economic cost [6]. The descriptive epidemiology of childhood cancer also varies in accuracy. In countries where registration of childhood cancer cases is almost complete, the epidemiology of childhood cancer shows little or no increase in incidence of new cases per annum [7]. By contrast, in some countries, for example some Eastern European countries [8] and India [9], registration is poor and compromises research on trend analysis and disease aetiology. In low-income countries, it has been suggested that hospital-based registries could be developed [10].

Although there is considerable interest and active research on the chemopreventive potential of NSAIDs, this has yet to be directed towards childhood cancers to the same extent as adult malignancies. In this paper, hypotheses are presented for the first time in the expanded form that salicylate might exert benefits against childhood cancer. These hypotheses build on previous work [11] and possible future lines of research are identified. To offer a broader context, some aspects of childhood cancer aetiology are initially considered. Outside of the scope of this paper, however, is the proposal and broader debate that salicylate might be reclassified as vitamin S [12].

## Aetiological aspects of childhood cancer

With the exception of retinoblastoma (RB), where more than 40% of cases are due to an inherited mutation in the RB tumour suppressor gene [13], as well as in limited cases of Wilms tumour, Hodgkin’s lymphoma and neuroblastoma, there is no clear evidence for a familial predisposition to childhood cancers [14–16]. This would in turn suggest a prominent role of modifiable risk factors in the aetiology of childhood cancer.

One such risk factor might be the exposure to infections. There is a hypothesis for an infectious aetiology for acute lymphoblastic leukaemia (ALL) but not for acute non-lymphoblastic leukaemia (AnLL) or for solid tumours such as those of the central nervous system (CNS) and neuroblastoma [17]. More generally, it is known that certain infections can predispose to cancer [18], such as human papillomavirus and the hepatitis virus. Vaccination programmes against these viruses are helpful to reduce the risk of cervical and liver cancer, respectively. Perhaps aspirin may also influence the risk of viral-induced cancers such as those caused by the Epstein-Barr virus [19] and Cytomegalovirus [20] by inhibiting cell invasion and exerting anti-viral effects, respectively.

Whilst recognizing the complexity of childhood cancer as a group of diseases, coupled to the difficulties of differentiating association from causation, two further modifiable risk factors are briefly discussed.

### Maternal exposure to risk factors

Based on a study in the United Kingdom, socio-economic status does not appear to be a determinant of ALL in children [21]. This is potentially important since socio-economic status may be predictive of maternal lifestyle. Although much has been documented with regard to maternal diet, smoking, alcohol consumption and recreational or prescription drug use during pregnancy, there is no consistent evidence to support a link with any of these factors and childhood leukaemia [22].

Maternal exposure to solvents could further be investigated for ALL but evidence to date is limited given that it is based on self-reported data [23]. As a more general point, the adverse health effects linked to maternal exposures include foetal death, birth defects, being small for gestational age, pre-term birth, clinically overt cognitive, neurologic and behavioural abnormalities, subtle neuropsychological deficits, childhood cancer, asthma, other respiratory diseases and acute poisoning [24].

So, there is a broader challenge of correlating exposure to outcome for child health [25]. Expert groups faced with evaluating epidemiological evidence of potential causal relationships repeatedly encounter problems in summarizing the available data.

### Vitamin influence on childhood cancer risk

Folic acid, vitamin B, is used in several countries as part of commercially available supplements and fortified foods for disease prevention [26]. Supplementations of folic acid to pregnant women have a beneficial effect in reducing the risk for neural tube defects in the offspring. There is evidence suggesting that folic acid supplementation also may reduce the risk of certain childhood cancers. Folic acid fortification in a Canadian study was associated with a significant 60% reduced risk for neuroblastoma development, whereas the incidence of ALL and hepatoblastoma remained unchanged [27].

Another study reported that maternal use of folic acid, both pre-conception and peri-conception, reduced the risk of brain tumours in the offspring. This may suggest that perhaps folic acid supplementation might prevent the development of cancers in a site-specific manner [28]. Moreover, pre-conception use of maternal multivitamin supplements containing folic acid demonstrated protective effects for childhood leukaemia, paediatric brain tumours and neuroblastoma [29]. Partly in contrast, a study from Germany showed that maternal use of multivitamins, folate and iron supplements reduced the risk of non-Hodgkin’s lymphoma and perhaps leukaemia, but not tumours of the CNS. This study also demonstrated an associated increased risk for neuroblastoma in the offspring [30].

It has further been suggested that vitamin D has potential anti-cancer activity and vitamin D status is sub-optimal in many children in North America [31]. There is also a hypothesis of an increased risk of ALL following vitamin K given to newborns [32]. These influences, although questions remain, highlight the possible role of dietary components in the aetiology of childhood cancer.

## Hypotheses that salicylate might protect against childhood cancer

The evidence on the factors of childhood cancer is far from complete and there are risk-significant unresolved questions and gaps in knowledge. Given this, there is capacity for additional factors that influence childhood cancer risk to be put forward. It is on this basis that there is scope to suggest hypotheses that salicylate might help reduce the risk of childhood cancer.

Aspirin is now rarely used in children because of an association with a condition known as Reye’s syndrome [33]. It has been suggested that the reduced use of the medicine in children could be a factor in increasing childhood asthma [34].

The rare use of aspirin in children, which *per se* may have negative consequences, focuses a ***prima facie*** hypothesis on salicylate and childhood cancer on two factors. Firstly, salicylate exerts apoptotic effects on a broad range of cancer cell lines and NSAIDs inhibit the development and progression on pre-clinical ***in vivo*** models of neuroblastoma and medulloblastoma [35–37].

Secondly, salicylate is a natural component of fruits and vegetables so it is consumed in the diet [38]. Salicylate plays an important role in protecting tissues from disease and damage through the induction of apoptosis and in the inflammatory resolution phase. It therefore follows that those fruits and vegetables grown in natural conditions where they are exposed to the environment and pathogenic challenge express the highest levels of salicylate in their tissues [38]. A related hypothesis is that humans are becoming increasingly salicylate deficient [39] due to changes in food production and consumption patterns [40].

There has been interest in the vegetarian diet and reduced cancer risk [41]. It would therefore appear that the pharmacological properties of salicylate from pharmaceutical or dietary sources have relevance to human health and disease. Evidence extending over several decades demonstrates that salicylate exerts a number of effects on prostaglandin pathways [42]. More recent evidence suggests that these effects appear to be dependent, at least in part, on the intra-cellular oxidative conditions [43]. Salicylate might also exert anti-inflammatory effects through non-COX mechanisms [44]. For example, there is evidence that salicylate exerts its anti-inflammatory action in part through inhibition of the extracellular signal-regulated kinase (ERK) pathway. This is potentially important given that tumour necrosis factor-α can induce the ERK pathway [45].

There is also evidence that certain metabolites of salicylate may inhibit COX-2-dependent prostaglandin E_2_ formation at sites of inflammation [46]. Further research would appear to be warranted to clarify the anti-inflammatory pathways of salicylate and metabolites [47]. Recently, acetylation of COX-2 by low-dose aspirin was shown to redirect the catalytic activity of COX-2 away from generating pro-inflammatory prostaglandins and thromboxanes towards the production of lipoxins. The COX-2 enzyme with this modification remains catalytically active [48]. In contrast to prostaglandin E_2_, which has proven pro-carcinogenic effects, one of the major end products of acetylated COX-2 is lipoxins (15-epi-lipoxin A_4_), which have anti-tumourigenic effects [49]. In addition, other lipid mediators such as resolvins and protectins, important in the resolution phase of inflammation, are also produced by COX-2 and lipoxygenase enzymes from the omega-3 fatty acids eicosapentanoic acid (EPA) and docohexanenoic acid (DHA), which could explain some of the anti-carcinogenic properties of these compounds [48,49]. It is possible that the ingestion of salicylate exerts multiple effects, directly as a parent compound and also with respect to the metabolites produced. Research on these effects in human health and disease might help to identify new therapeutic targets for cancer control, both in children and adults.

### Effects of NSAIDs on pre-clinical models of solid childhood tumours

Several studies have shown that different NSAIDs inhibit the growth of some solid childhood cancer cells ***in vitro***, notably neuroblastoma, medulloblastoma including primitive neuroectodermal tumours (PNET) osteosarcoma and rhabdomyosarcoma [35–37,50–58]. Furthermore, both the COX-2-specific NSAID celecoxib and the dual COX-1/COX-2 inhibitor diclofenac significantly inhibit neuroblastoma and medulloblastoma growth in animal models [35–37].

Moreover, prophylactic treatments with celecoxib inhibit or prevent the development of neuroblastoma xenografts in rodents [54]. Celecoxib also significantly enhanced the effects of common chemotherapeutic drugs used in the treatment of neuroblastoma and medulloblastoma [35–37]. Medulloblastoma, PNET and neuroblastoma primary tumours express high levels of COX-2 and microsomal prostaglandin E synthase-1 (mPGES-1), the two enzymes responsible for converting arachidonic acid, a dietary fatty acid and precursor to eicosanoid synthesis, to prostaglandin E_2_. Compared to non-malignant nervous tissue, neuroblastoma, medulloblastoma and other childhood nervous tumours contain increased levels of arachidonic acid [60,61].

The presence of high levels of COX-2 and mPGES-1 in these tumours may therefore generate a pathogenic environment for the increased production of pro-carcinogenic prostaglandins. It has also been shown that prostaglandin E_2_ increases proliferation of medulloblastoma cells and patients with malignant brain tumours have significantly higher concentrations of prostaglandin E_2_ in their tumours and plasma compared to patients with benign brain tumours or non-cancer control patients [59–61]. Moreover, the concentration of prostaglandin E_2_ in plasma decreases following neurosurgical resection of both malignant and benign brain tumours [61].

Taken together, these results suggest that dietary salicylate might be beneficial in the prevention of at least a subset of childhood cancers.

### Salicylate prevention of first and subsequent childhood cancers?

Whilst recognizing that there may be some risks with ingesting high-levels salicylate during pregnancy [62], women may be exposed in two broad ways, which confer benefit. As suggested previously, the first of these is through dietary sources. The second is that some women might take aspirin, and there is evidence that in some cases of pre-eclampsia, low-doses of the medicine might be beneficial and increase birth weight [63]. This is potentially important ***per se*** given that low birthweight has been associated with numerous health problems during life [64].

One possible line of future research is therefore to understand the levels and effects of maternal exposure to salicylate on the risk of childhood cancer. More generally, such research could also examine maternal exposure to salicylate and other aspects of childhood health.

Maternal salicylate would also be expected to be secreted through breast milk. The effect of breastfeeding on childhood cancer risk is uncertain and increasing breastfeeding from 50% to 100% in the population has been estimated to prevent perhaps 5% of cases of childhood acute leukaemia or lymphoma at most [65]. Another consideration is the evidence that childhood fruit consumption may have a long-term protective effect on cancer risk in adults. Further prospective studies, with individual measures of diet, are required to further elucidate these relations [66].

There is evidence of a sustained increased risk of second malignancy in those treated for primary cancer, especially those diagnosed in childhood [67]. A study of salicylate levels in childhood cancer patients might therefore be a valuable research avenue. Such studies might measure salicylate levels non-invasively using readily available bodily fluids, such as urine. Perhaps dietary salicylate, particularly high in fruits and vegetables grown under organic conditions, could help reduce the risk of second malignancy. This could be tested experimentally through randomized trials with the active intervention being exposure to salicylate through fruits and vegetables.

Furthermore, salicylate might be an endogenous protective agent against cancer. There is evidence that humans have the ability to synthesize salicylate [68]. Salicylate deficiency or impaired metabolic pathways leading to synthesis might therefore be a factor in childhood cancer risk. In rare cases, however, some individuals experience salicylate intolerance [69]. A study of cancer risk in intolerant individuals might also be a productive research line. If salicylate does confer protection then intolerant individuals might have an increased risk of cancer.

Furthermore, laboratory-based experiments would also be helpful to increase knowledge and understanding of the effects of salicylate on childhood cancer.

## Some contemporary issues in childhood cancer

Research evidence suggests that the continuing needs of childhood-cancer survivors include the provision of information of the later effects of treatment and self-care [70]. With respect to self-care, this might include tailored exercise programmes given that reduced physical activity is often a feature of childhood cancer, during and after treatment [71].

Across the world, childhood-cancer survival rates are increasing, for example one study from Singapore suggests that approximately 60% of patients overall survive more than two years [72]. Social aspects of surviving childhood cancers, such as impact on relationships, possibly highlight the need for longer support services to be offered [73]. Such psychosocial support might also be delivered to family members of childhood-cancer survivors [74].

Efforts to improve the cosmetic aspects of childhood cancer treatment, for example following irradiation of facial tissues, are also being developed and include the grafting of adipose tissue into affected areas [75]. Quality-of-life considerations in childhood cancer are also important and may differ, depending on the specific pathology. For example, evidence suggests that survivors of leukaemia have better quality of life than those with brain tumours [76].

Efforts to minimize the deleterious effects of childhood cancer treatment are also progressing [77]. Further research on the long-term health sequelae of surviving childhood cancer also appears warranted. There is evidence that irradiation, whole body or abdominal, can lead to an increase risk in developing type-2 diabetes mellitus [78] and, in females, complications in pregnancy have been described [79]. Increased risk of several long-term chronic health conditions, such as musculoskeletal disease, has been also observed in survivors of neuroblastoma [80]. Growth-hormone deficiency problems have also been described in childhood-cancer patients receiving cranial radiotherapy [81].

The scope for research and development on childhood cancer is considerable. This ranges from basic scientists continuing to develop pre-clinical cancer models, for example as demonstrated by work on hepatoblastoma [82], through to emergency department staff [83] as they may have opportunities to identify disease. The value of childhood cancer treatment being delivered by multidisciplinary teams appears important [84], especially when there is a high degree of distress within the family [85].

## Closing remarks

Many lines of contemporary research and development are active in the field of childhood cancer research. Given the extensive, although not conclusive, evidence base supporting a potential benefit of aspirin against cancer, studies on salicylate and childhood cancer risk appears worthwhile. Further research, of which several lines are identified in the paper, is recommended.
